# An effective environmental enrichment framework for the continual improvement of production animal welfare

**DOI:** 10.1017/awf.2023.5

**Published:** 2023-02-14

**Authors:** Peta S Taylor, Peggy Schrobback, Megan Verdon, Caroline Lee

**Affiliations:** 1 School of Envrionmental and Rural Science, University of New England, Armidale, 2351, NSW, Australia; 2 CSIRO, Agriculture and Food, St Lucia, QLD, Australia; 3 Tasmanian Institute of Agriculture, University of Tasmania, Burnie, TAS, Australia; 4 CSIRO, Agriculture & Food, Locked Bag 1, Armidale, NSW 2350, Australia

**Keywords:** animal welfare, economics, pleasure, positive affect, practicality, quality of life

## Abstract

Substrates and objects are provided to farm animals on the assumption that they improve animal welfare by enriching the environment, but these often fail to consider the extent to which an environmental enrichment (EE) improves animal welfare, if at all. Furthermore, there are numerous definitions of EE, each with a unique expectation. If expectations of animal welfare improvement are set too high, industry uptake may be thwarted, but if thresholds are set too low it will not result in meaningful improvements to animal welfare. We propose an EE framework based on revised definitions of EE that reflect improvements to various components of animal welfare: (i) pseudo-enrichment; (ii) EE for meeting basic needs; (iii) EE for pleasure; and (iv) EE for positive welfare balance. This framework requires short- and long-term assessments to determine the impact of the EE, although many are lacking in the production animal literature. Redefining EE with a focus on specific animal welfare outcomes will assist producers in identifying the optimal EE for their enterprise. Subsequently, we encourage dialogue between farmers, researchers and industry stakeholders when designing environmental enrichment programmes. This framework is a science-based tool that can be used to inform the development of clear EE assessment protocols and requirements for animal welfare legislation, assurance programmes and industry. This evidence-based framework ensures that the focus is on the *outcome* of EE programmes rather than the *intent.* Importantly, this framework has the flexibility to adapt even as baseline environments evolve, ensuring the continual improvement to production animal welfare.

## Introduction

The provision of environmental enrichments in captive animal housing is becoming increasingly prevalent, largely due to an increasing public awareness and concern for animal welfare (Grunert *et al.* 2018; Alonso *et al.* 2020), and the perception that enrichments improve animal welfare (Schütz *et al.* 2020). Although the provision of enrichment is not legislated worldwide, recent years have seen the regulation of enrichment by welfare accreditation schemes, for example, RSPCA-approved farming schemes (RSPCA Australia 2021). Despite the good intentions of supplying environmental enrichment, some enrichment programmes have no effect on animal welfare, and in some circumstances can even negatively impact welfare. For example, increased competition and aggression when straw is provided as a limited resource to beef cattle (*Bos taurus*) and sows (*Sus scrofa*) (Pelley *et al.* 1995; Stewart *et al.* 2008) or increased mortality after the provision of environmental enrichment items to meat chickens (*Gallus gallus domesticus*), possibly due to an inability to find feed and water in the more complex environment (Gordon & Forbes 2002). Furthermore, there is a risk that requirements to provide ‘enrichment’ may simply ‘tick a box’ rather than lead to actual animal welfare improvements, i.e. the outcome may not improve animal welfare, but the item would meet ‘enrichment’ criteria from a legislative point of view. This could be misleading to consumers, resulting in distrust from the public towards industry attempts to improve animal welfare, and development of negative perceptions surrounding the benefits of enrichment provision from producers.

The subjective nature of the term ‘environmental enrichment’ also poses issues for researchers, industry and public expectations. The term, enrichment, has been used to describe the provision of a resource or environment that either prevents suffering (i.e. improves biological functioning [Newberry 1995]), provides an environment beyond suffering (Boissy *et al.* 2007) or refers to a gold standard species-specific environment providing “optimal psychological and physiological well-being” (Shepherdson 1998). The various degrees of improvements to animal welfare outlined by each of these definitions sets a different expectation as to what an enriched environment might look like. Consequently, the definition of ‘environmental enrichment’ may set the requirements of ‘enriched’ at too high a level, which disincentivises industry uptake, or too low a level to achieve continual, meaningful, improvements for animal welfare.

The term ‘environmental enrichment’ without reference to the animal welfare outcome may result in the expectations of various stakeholders not being met. For example, if the goal of environmental enrichment is to improve animal welfare, how much improvement to animal welfare is required before an environment is considered effectively ‘enriched’? Measuring and labelling relative animal welfare improvements after the provision of enrichment could encourage continual improvements to welfare through environmental enrichment. Even as societal acceptance of current environments evolves and the quality of standard/baseline environments shift, terminology that defines enrichment as the *relative* improvements to animal welfare will remain relevant.

In various parts of the world, the provision of enrichment is encouraged but is not a legal requirement (e.g. pigs and poultry in Australia [CSIRO 2002, 2008]). There are no available data on how many Australian farms are currently providing enrichment to their livestock or information regarding the (perceived or real) barriers that are preventing implementation of enrichment programmes. Although cost has been reported as a significant barrier to providing enrichment to pigs by farmers in the UK (Peden *et al.* 2021). An enrichment is unlikely to be used if the people managing livestock perceive it as costly with no clear return (i.e. no clear benefits to welfare, production or social licence). Categorising animal welfare outcomes after the provision of enrichment, and aligning these outcomes with economics, may assist the dialogue between producers, researchers and regulatory bodies and subsequently increase the provision of effective enrichment to livestock.

In this study, we propose a framework to re-define and re-categorise environmental enrichments based on the outcomes for animal welfare. We propose four categories of environmental enrichment to accurately reflect the outcome of animal welfare improvements, regardless of the baseline environment. The framework is based on Dawkins’ (2008) definition of animal welfare and the Rault *et al.* (2020) definition of positive welfare state and balance and builds upon the statement by Newberry (1995) that “enrichment implies improvement” as well as the approach to welfare assessments of Fraser (2006) and Edgar *et al.* (2013). Categorising the term enrichment in this way will improve clarity, expectations and ultimately the impact of environmental enrichment programmes on animal welfare.

We consider that enrichment programmes require other multi-stakeholder considerations before they can be considered ‘effective’ to implement. This is based on the premise that to truly be ‘effective’, an enrichment needs to not only improve animal welfare but must also be practical and economical for industries to apply (van de Weerd & Day 2009). Enrichments that do not meet these criteria will likely not be implemented, regardless of whether they improve animal welfare or not. For example, if an enrichment has a positive effect on animal welfare but there are currently no possible waste management solutions (such is the case for straw in some commercial piggeries), it cannot feasibly be implemented and therefore will not be effective. Similarly, if an enrichment is practical and low in cost to implement but does not positively impact animal welfare it also cannot be considered effective. Over time, research, development and market change may overcome such economic and practical challenges but if not (and in the meantime) such environmental enrichments will not be implemented and therefore will not be effective. In other words, the effectiveness of the enrichment is solely related to the animal welfare *outcome* rather than the *intent.* Thus, our framework includes three components: animal welfare outcomes; economics; and practicality.

## Materials and methods

A literature review of environmental enrichments and an industry survey regarding the practicality of various enrichments formed the basis of the framework (Taylor & Lee 2021). Five intensively housed species were included in the review: laying hens; meat chickens; farrowing and gestating sows; feedlot beef cattle; and feedlot sheep (*Ovis aries*). Physical, social, nutritional, cognitive and sensory environmental enrichments were included. Welfare outcomes of each enrichment were recoded into a data file including animal welfare outcomes in relation to physical and mental health, abnormal and natural behaviours, impacts on production and animal preferences for, and utilisation of, enrichments (classifying each welfare indicator as either improved, no change or a negative impact). Collated data are presented on the ‘Enriching Australian Livestock’ website (UNE & England 2022). Enrichments that provided evidence of improvements to animal welfare were included in an online industry survey aimed at assessing the practicality of each enrichment.

A survey document to assess the practicality and economic feasibility of enrichments was developed and distributed through an online survey platform (Qualtrics XM, Provo, UT, USA). The online survey was distributed to stakeholders of the five aforementioned livestock industries through the National Animal Welfare RD&E strategy (see supplementary material for full survey). A representative from each livestock industry (n = 5) circulated the survey to their networks through email correspondence. Additionally, all other members of the National Animal Welfare RD&E strategy were asked to circulate the survey to their networks (n = 27 organisations [NAWRDE Strategy 2019]). Multiple follow-up requests to participate were sent to the aforementioned industry representatives via email from the researchers and the executive secretary of the National Animal Welfare RD&E Strategy. The survey included questions regarding the participant (i.e. stakeholder category; producer [n = 8]; industry representative [n = 2]; welfare officer [n = 1]; veterinarian [n = 5]; non-government organisation [n = 2]; other [n = 8]), if specific enrichments had been, or were, utilised, whether implementation was practical and questions relating to the perceived benefits and barriers of environmental enrichment. Collection of survey data was approved by the University of New England Human Ethics Committee (HE20-223) and was conducted between February and April 2021. Survey responses were low. A total of 26 stakeholder responses from the five industries were recorded (n = 11 chicken meat; n = 2 cattle/dairy; n = 8 pork; n = 1 sheep; n = 4 egg). However, data are presented in this paper to place the proposed enrichment framework within the context of industry perspectives.

## Survey response

**Figure 1.**
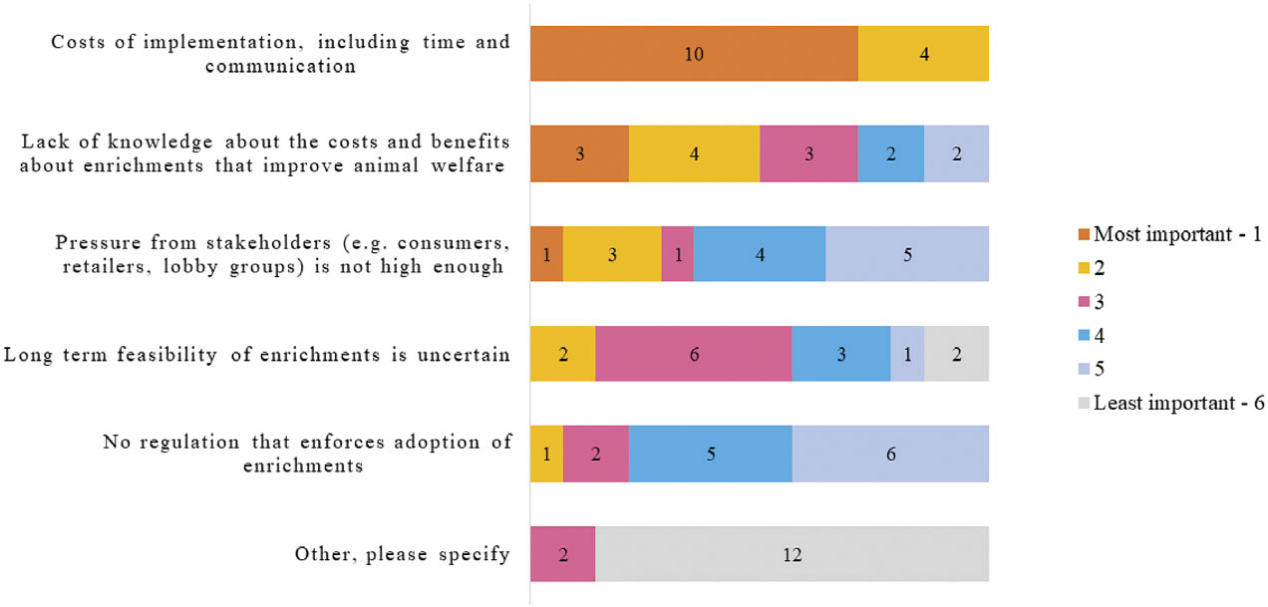
Industry stakeholder responses (n = 14) when asked to rank the barriers to implementation of environmental enrichments from 1 (most important) to 6 (least important).

Survey response was low (n = 26) and only 14 of the respondents completed the entire survey. Therefore, results presented here are pooled (i.e. all responses from the various livestock sectors are presented together). The greatest perceived barrier to implementing environmental enrichment was cost; ranked the number one barrier by 72% (n = 10) of respondents (Figure 1).

**Figure 2.**
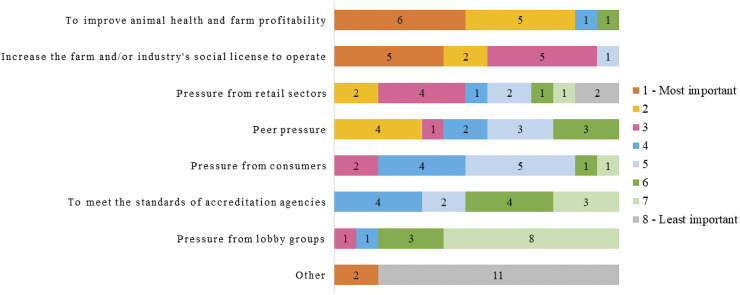
Industry stakeholder responses (n = 13) when asked to rank motives to implement environmental enrichments from 1 (most important) to 8 (least important).

Improving animal health and farm profitability and increasing social licence were ranked as the greatest motivation to provide environmental enrichment on farms (Figure 2).

## Proposed framework

**Figure 3.**
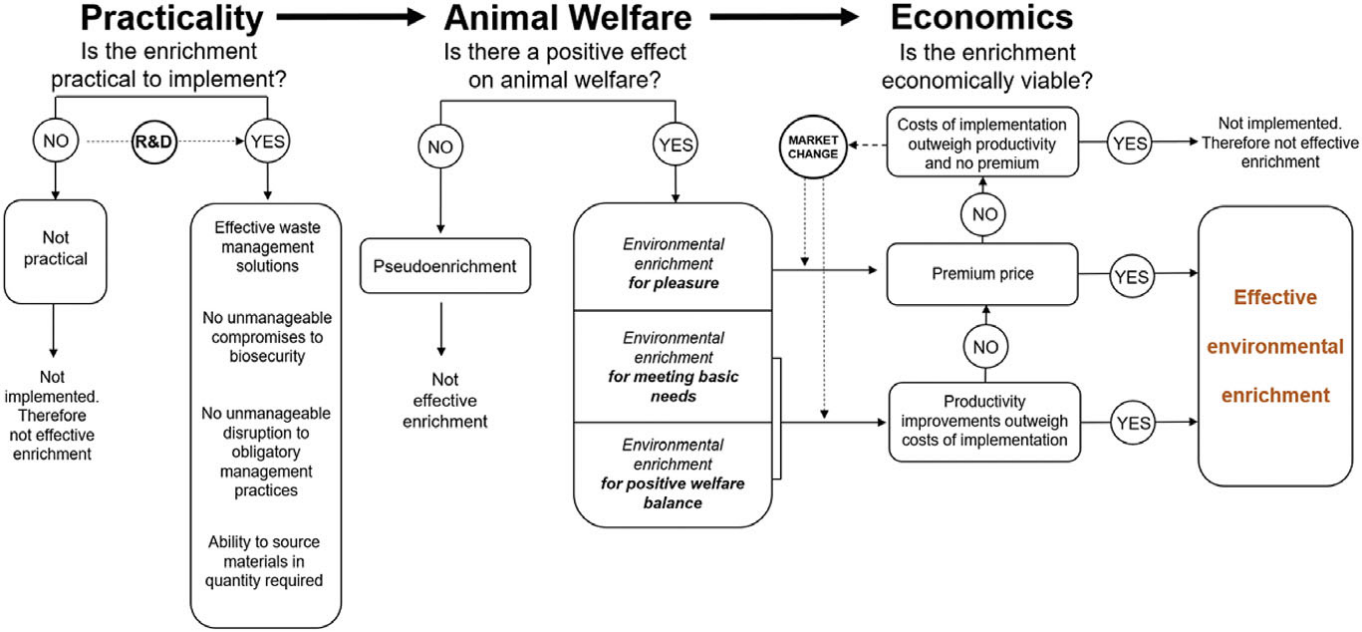
Framework for effective environmental enrichment for livestock species outlining three major components that must be considered: practicality; animal welfare outcomes; and economics. Dashed arrows indicate opportunities for development and change providing solutions to economic and practicality barriers. R&D indicates the potential of innovation that may result in improvements in practicality over time. Market change indicates market dynamics (e.g. price, cost) that may lead to economic benefits outweighing the costs of implementing an enrichment. Pseudo-enrichment refers to proposed environmental enrichment resources that do not improve animal welfare.

The proposed framework includes welfare outcomes, economics and practicality (Figure 3). Each component will be discussed individually before being presented and considered together. Re-categorising each environmental enrichment based on the welfare outcomes aims to: (i) ensure the term ‘environmental enrichment’ is reserved for situations only when animal welfare is improved even as baseline environments evolve (i.e. excludes pseudo-enrichments that may have good intentions but no positive implications for animal welfare); and (ii) link the characteristic of animal welfare improvements to economic returns to provide clear incentives to producers to provide environmental enrichment proven to improve animal welfare.

### Animal welfare outcomes

For an environmental enrichment to be effective it must improve animal welfare. The nature of the improvement may be complex due to, for example, potential animal welfare trade-offs (e.g. negative health impacts but improvements to positive affect — see section on risk below), and the method of welfare assessment. The optimal approach to assess animal welfare has been debated but it is now well accepted that there is not one single indicator of animal welfare and, hence, welfare is best assessed using a combination of indicators (Fraser *et al.* 1997; Duncan 2005; Fraser 2008; Hemsworth & Coleman 2011). We consider short- and long-term effects and ask if the provision of a specific environmental enrichment reduces suffering (i.e. ‘enrichment for meeting basic needs’; evident by fewer injuries, less pre-clinical and clinical disease and normal biological functioning), and/or if the enrichment is something that the animal wants beyond basic needs (i.e. ‘enrichment for pleasure’; evident by preference, behavioural demand, long-term utilisation of enrichments and ‘enrichment for positive welfare balance’; long-term effects of positive experiences that outweigh negative welfare experiences, such as positive mood and stress resilience; Table 1). We refer to basic needs as both ultimate and proximal physiological and behavioural needs that when thwarted result in poor health, frustration or stress. The approach implies the nature of the improvements to animal welfare, whilst acknowledging that providing an animal with what it wants and likes can provide positive experiences even when not all of its basic needs are met (see Rault *et al.* 2020).

**Table 1.** Proposed re-classification of environmental enrichment provided to animals based on both short- and long-term assessments of welfare indicators

Short term assessments of welfare		Long term assessments of welfare		
Biological functioning	Positive hedonistic states	Sustained use	Positive mood or stress resilience	Proposed terminology
↔	↔	↔	↔	Pseudo-enrichment
↔	**↑**	↔	↔	Pseudo-enrichment*
**↑**	↑ or ↔	↑ or ↔	↔	Enrichment for meeting basic needs
↔	**↑**	**↑**	↔	Enrichment for pleasure
↑ or ↔	**↑**	**↑**	**↑**	Enrichment for positive welfare balance

↑ Indicates improvement after the provision of enrichment.

↔ Indicates no improvement to welfare indicator after the provision of enrichment.

Pseudo-enrichment refers to proposed environmental enrichments that do not improve biological functioning, provide sustained pleasure or result in a positive welfare balance.

* This item may be beneficial if it is included in an enrichment programme or rotated throughout an animal’s life, however when provided in isolation does not improve the welfare of the animal.

Barren environments and resources that do not contain a specific resource that an animal needs, can lead to suffering. Suffering can disrupt biological functioning which will be evidenced by compromised fitness traits, such as increased morbidity and mortality or impaired growth/body condition and reproduction (Broom & Johnson 1993; Moberg 2000; Hemsworth & Coleman 2011). When an animal is provided with an environmental enrichment that meets a need, the prolonged stress responses and subsequent impacts on health and biological functioning will improve (Fraser & Duncan 1998). As such, this component of the framework asks the question, does the enrichment prevent, or ease, animal suffering? Inclusion of ‘easing’ of suffering into this component builds on the Newberry (1995) definition of enrichment as ‘something provided to an animal that is biologically relevant and prevents suffering’ and supports continued improvements in animal welfare. Of note, here we focus on (relatively) short-term effects/assessments (days to weeks; Table 2) after the provision of an enrichment, not immediate acute stress responses (minutes to days) induced by environmental enrichment (i.e. some novel objects) or long-term assessments that improve welfare in the long-term, such as increased stress resilience (Zulkifli & Siegel 2019).

**Table 2.** Proposed assessments for each proposed environmental enrichment (EE) classification using science-based indicators of animal welfare. Short-term refers to days, weeks or months depending on the species and long-term refers to the whole life or production cycle of the animal

Proposed EE classification	Welfare improvements	Welfare indicators
Environmental enrichment for meeting basic needs	Short-term improvements to biological functioning and health which may, or may not, be accompanied by short-term positive hedonistic experiences	Reduction in injuries & lameness Reduction in pre-clinical and clinical disease Improved biological functioning
Environmental enrichment for pleasure	Short-term positive hedonic experiences *and* long term sustained use Enrichment does not improve biological functioning. In some cases, there may be an increased *risk* to health (i.e. prevenance or severity of injury, disease or mortality).	Sustained use of enrichment Preference Strong motivation to access Secretion of neurotransmitters associated with positive affect (i.e. dopamine and opioids)
Environmental enrichment for positive welfare balance	Accumulation of positive hedonic experiences and reduction in negative affective experiences that result in a positive welfare balance and long-term welfare benefits beyond immediate survival	Stress resilience, increased flexibility and ability to adapt to challenge Positive moods (i.e. cognitive bias)

In agreement with the statement that enrichment should improve welfare beyond the absence of suffering (Boissy *et al.* 2007), the proposed framework also considers both short-term positive hedonic experiences and long-term accumulative effects of positive hedonic experiences that outweigh negative experiences (Table 1). This question provides insight into the positive experiences that environmental enrichment can provide. Whether an environmental enrichment is something that an animal wants can be determined by assessing preferences for enrichments, motivation to access specific enrichments (behavioural demand) and use/interaction with the environmental enrichment over time (Table 2). Evidence suggests that what an animal *wants* (i.e. a specific environmental enrichment, or chooses one environmental enrichment over another) reflects what an animal *likes* and the associated experience of positive affective state (emotions), such as pleasure when it is obtained (Fraser & Duncan 1998; Fountain *et al.* 2020; Mendl & Paul 2020). However, there are many factors that must be considered with assessing positive hedonistic experiences through motivation and preference, such that the choices animals make can change over time. For example, cattle prefer pasture rather than a feedlot environment in the evening but prefer a feedlot environment in the morning; preferences are related to lying and feeding behaviour, respectively (Lee *et al.* 2013). Furthermore, animals may become habituated to an enrichment which would be indicated by a decline in environmental enrichment utilisation over time (Guy *et al.* 2013). This suggests that preferences, motivations and utilisation of enrichments should be monitored over the entire time-frame of interest, i.e. the whole production cycle or for the period of time that the environmental enrichment is allocated. If habituation occurs, our framework suggests that this enrichment on its own is not effective, although it may be effective when combined or rotated with other environmental enrichment items in a programme designed to maintain animal interest and use throughout their lifespan. For example, rope, straw and wood on a chain is more effective at improving sow welfare when provided on a rotation compared to consistent and continuous access (Roy *et al.* 2019) and novel objects that are not biologically relevant when rotated can improve chicken welfare (Altan *et al.* 2013).

Our proposed framework includes short- and long-term assessments of animal welfare after the provision of potential enrichment items or programmes to understand. This approach more fully elucidates the nature of the improvements to animal welfare which enables the type of enrichment to be categorised (Table 1). Our framework then considers each category of enrichment in the context of economics. We argue that capturing the nature of the improvement to welfare allows clearer links between improvements to animal welfare and economics and, subsequently, this approach will increase uptake by producers and provide clarity to consumers looking to make informed buying decisions.

### Characteristics of animal welfare outcomes

A prologue to this component of the framework acknowledges that we do not propose that animals housed in environments provided with effective environmental enrichments that they want and/or need are living ‘beyond basic needs.’ Rather, we provide a framework that can be used in a variety of ‘standard’ environments (noting that what is considered to be ‘standard’ constantly changes and evolves) to determine the magnitude of change of animal welfare after an environmental enrichment item/programme is provided; either enriching an animal’s environment by reducing suffering (i.e. environmental enrichment for meeting basic needs), providing pleasure or improving overall balance of positive welfare. Although our framework could suggest that environmental enrichments that reduce suffering are a lower tier than environmental enrichments that provide pleasure, we do not suggest that one is more important than the other. This approach acknowledges that enrichments that reduce suffering in some present-day environments may not be beneficial in the future (i.e. if the quality of standard housing environments improve) but also provides an avenue to provide environmental enrichments that improve animal welfare without prescribing unobtainable criteria for producers (i.e. provision of pleasure even if basic housing does not meet all of the animal’s basic needs). Thus, the framework provides a fluid threshold to determine ‘enriched environments’ which promotes continual improvements to production animal welfare.

## Proposed environmental enrichment classifications

### Pseudo-enrichment

*Pseudo-enrichment* is a term devised by Würbel and Garner (2007) referring to enrichments that do not improve rodent welfare in research and laboratory captive environments. We apply this terminology to refer to proposed environmental enrichment items and programmes that do not improve physical health and biological functioning, nor is there evidence that the enrichment is wanted by the animal. Not only is the animal unable to engage in intrinsically valuable experiences but the environment is lacking specific opportunities that an animal needs, resulting in chronic stress. As such, any enrichment in this category must never be considered effective environmental enrichment.

### Environmental enrichment for meeting basic needs

*Environmental enrichment for meeting basic needs* indicates that the enrichment provides something that improves biological functioning. Enrichments that improve basic needs (i.e. physical health or biological functioning) focus on the ease of suffering. As such, they may be prescribed as standards in legislation or eventually be included in ‘standard housing’ environments. Enrichments in this category may simply reduce boredom in a barren environment, rather than provide something specific that an animal wants and, therefore, the outcome on animal welfare may not be directly transferable across production systems, enterprises or other environments. We acknowledge that positive affective states are likely experienced when a basic need is met, however this category excludes any environmental enrichments that result in long-term benefits for positive welfare balance (i.e. stress resilience). Theoretically, this category of enrichment may be classified as *environmental enrichment to reduce suffering* however this terminology may be too emotive for producers, industry or consumers, and therefore may limit uptake and support. Depending on the standard baseline, environmental enrichments provided in this category *may* provide animals with ‘a life worth living’ according to the Farm Animal Welfare Committee (2009). Although, it must be noted that environmental enrichments are not the only method to provide an animal with ‘a life worth living’ or ‘a good life’ — we restrict our discussions to environmental enrichments here, for a broader perspective see Farm Animal Welfare Committee (2009).

### Environmental enrichment for pleasure

*Environmental enrichments for pleasure* are environmental enrichment items or programmes that provide the animal with something that it wants but does not improve health or biological functioning (i.e. it is something that an animal wants, but does not need) nor does it result in a positive welfare balance. Short- and long-term assessments are required to identify these environmental enrichments, such that use of the item (i.e. pleasure) must be sustained. We acknowledge that such environmental enrichments provide positive hedonistic experiences (i.e. reward and pleasure) which may be independent to experiences of negative affect (i.e. fearfulness, anxiety). Therefore, it is important to note that environmental enrichments in this category do not impact survival or biological functioning, rather they exceed basic needs and may be termed ‘luxury’ items (Fraser & Duncan 1998). The positive experiences provided by this environmental enrichment are *not* adequate to outweigh the negative experiences (i.e. hence there is evidence of positive hedonic experiences but no evidence of long-term positive welfare balance such as increased stress resilience or positive mood). This category may be particularly important as baseline environments evolve (i.e. as resources for basic needs are considered ‘housing’ rather than ‘enrichments’). The importance of this category may also be apparent for environments that have limited options to provide enrichment to meet basic needs (i.e. space). As such, with this category of environmental enrichment we acknowledge that not all of an animal’s needs must be met for it to experience pleasure but experiencing pleasure is an important component of animal welfare, even if the positive experiences are not enough to result in an overall positive welfare balance.

Some environmental enrichments that enrich *for pleasure* can be associated with risks to physical health. This may seem counter-intuitive but, if the risk can be managed, the environmental enrichment can still benefit animal welfare. The provision of access to an outdoor range for chickens provides a good demonstration of this point. Some individuals are highly motivated to frequently access the outdoor range (Larsen *et al.* 2017), suggesting one or more components of the outdoor area is something that they want. However, there may also be negative consequences of range access such as the presence of predators and risks of disease (Campbell *et al.* 2020). Enrichments that provide animals with something that they want, but may cause additional stress or injury (e.g. through increased aggression) also fall into the category of environmental enrichment for pleasure with risk. For example, sows are highly motivated to utilise straw but providing straw in a rack (i.e. as a point source material) results in competition to access the enrichment with dominant sows delivering increased aggression to monopolise the resource (Elmore *et al.* 2011). Environmental enrichments for pleasure that are associated with increased risk require additional management inputs (and thus additional economic inputs) to reduce the associated risks. Of note, some risks may be too great to consider regardless of other positive welfare outcomes, for example, dilution of medications due to nutritional enrichments or string causing necrosis after being caught on legs or tongues (Schlegel & Brash 2015).

Enriching for pleasure does not improve biological functioning or long-term health (i.e. stress resilience) which are both associated with economic returns. Unless there is an economic return (e.g. premium price paid for free-range eggs), this enrichment may be impractical for the producer to implement and thus not effective, see section below on economics.

### Environmental enrichment for a positive welfare balance

*Environmental enrichment for a positive welfare balance* results in long-term accumulative effects of positive experiences for animals, such as that outlined by Rault *et al.* (2020) in their Vienna framework, suggesting that animals provided with this type of environmental enrichment have a positive welfare balance (i.e. positive experiences outweigh the negative experiences throughout their life). Environmental enrichments in this category provide hedonistic positive experiences to animals which are short-term experiences, but the accumulative effects of such positive experiences and/or a reduction in negative affective experiences result in improved stress resilience, increased flexibility and adaptability to stressors or positive moods. Of note, such outcomes may be achieved not just via accumulative experiences of pleasure, but also include the positive effects of agency and improved cognition associated with the provision of some environmental enrichments.

### Animal welfare assessments

Prior experience with an enrichment can impact the response to enrichment later in life. For example, piglets reared in barren environments and weaned into enriched environments show evidence of improved welfare. However, piglets reared in enriched environments that are weaned into barren environments have compromised welfare (i.e. less play and more belly-nosing), even in comparison to piglets both reared and weaned in barren environments (Oostindjer *et al.* 2011). Similarly, hen welfare can be compromised if adult production environments contain specific enrichments (i.e. perches) that are not available in the rearing environment (Hester *et al.* 2013). As such, the impact of environmental enrichment items and programmes must be assessed at multiple times in an animal’s production life. For our framework this includes short- and long-term assessments. Of note, the timing of such assessments will be species-specific; i.e. meat chickens lifespan is typically 4 to 6 weeks of age whereas feedlot cattle and laying hens typically live for over 1 to 1.5 years and may be kept in various housing (i.e. pasture to feedlot or rearing sheds to free-range sheds). Long-term welfare assessments measure positive welfare balance (i.e. stress resilience, mood) and pleasure associated with utilisation of the enrichment (ensuring no habitation occurs) and short-term assessments measure biological functioning (reproduction, growth, morbidity and mortality) which may be specific to one part of an animal’s life (i.e. rearing environments or parturition).

### Economics

Once an enrichment has been shown to have positive outcomes for animal welfare, it must also be considered economical. In our survey, cost was ranked as the most influential barrier for enrichment implementation by industry stakeholders (Figure 1) despite the positive perception of enrichment for animal health and farm profitability (Figure 2). This relates to the nature of the welfare outcomes and cost of enrichment implementation (e.g. costs of material and labour). The cost of enrichment implementation will depend not only on the enrichment itself but also on the size of the enterprise, geographical location, and housing system and therefore will need to be calculated and considered for each application. We propose that the return on investment is reflected by the welfare outcome (discussed below) and should be considered when developing or revising minimum requirements for welfare policy and third-party quality assurance schemes.

Although production is not always associated with improvements to animal welfare, and *vice versa*, it is widely accepted that the physiological stress response can impact product quality and/or quantity (see Roberts 2004; Hemsworth & Coleman 2011). We propose that enrichments that lead to improvements to the basic needs of an animal and enrichments that provide a positive welfare balance, will be more likely to have a positive economic return due to improvements to productivity. For example, enrichments that improve biological functioning and/or health will impact fitness traits that are associated with increased productivity (i.e. reproduction and growth) and environmental enrichments resulting in an overall positive welfare balance (i.e. stress resilience and adaptation) are expected to reduce the effects of stressors (i.e. psychological or disease) diverting energy towards productivity and/or reducing morbidity and mortality. However, to be profitable, the improvement to biological functioning (and productivity) must be sufficient to offset the cost of implementation (e.g. materials, labour for placement and maintenance and disposal).

While there is always a chance that enrichments providing pleasure may show associated improvements to productivity, it remains an unlikely outcome. As such, the cost of implementation may outweigh the benefits unless the improvements to welfare are subsidised for the producer — particularly if the environmental enrichments are associated with risks to physical health that require additional management inputs/costs. A third party accreditation scheme (e.g. conformity audit according to an animal welfare standard and provision of certification labels) may also be needed to ensure that consumers receive the required signals (e.g. trusted labels) indicating that enrichments that improved animal welfare were implemented in the livestock production process. This is a further cost to livestock producers. Consumers may be required to pay a price premium for the livestock product they demand for this approach (e.g. enrichment implementation and standard conformity accreditation) to be commercially viable for livestock producers. Further investigation into the public perception of environmental enrichments for meeting basic needs, pleasure and positive welfare balance (however the improvements are phrased to the public) and the willingness to pay for such products is required to ensure that science-based improvements to animal welfare through the provision of enrichment is economically feasible for producers to implement.

An economic benefit-cost assessment of animal enrichment implementation should also consider the broader benefits for producers. For example, by implementing enrichments, businesses will likely gain access to a broader range of retailers in higher market segments which increasingly set private standards for animal health and welfare in the production process (Fraser 2006; More *et al.* 2017). The adoption of effective enrichments can also contribute to gaining/maintaining agri-businesses’ social licence to operate (Martin & Williams 2011). Lastly, livestock businesses may be rewarded for their implementation of enrichments through easier access to finance, since financial institutions are increasingly basing their investment decisions on sustainable production processes (Akomea-Frimpong *et al.* 2021) such as improved animal welfare for production animals.

### Practicality

We proposed that if an environmental enrichment is impractical to source, maintain or dispose it will never be implemented regardless of the impact on animal welfare. This includes providing an enrichment in a way that it can be effective, for example, ensuring enrichments are accessible to all animals (i.e. enrichment density) and/or provided at the appropriate time/age and frequency. The industry survey identified four main parameters regarding practicality that must be considered for the environmental enrichment to be effective (Table 3). These included how the direct or indirect waste from environmental enrichments could be removed, biosecurity risks, unmanageable interruptions to critical standard practices and the ability to source the environmental enrichment in the quantity and location required. Perceived issues of practicality must be addressed and considered by researchers and regulatory bodies when designing, investigating or prescribing enrichment programmes. Issues with practicality may be overcome with additional research and development (R&D), advances in technology or simply by providing scientific-based evidence to eliminate perceived impracticalities.

**Table 3.** Four main themes reflecting the practicality of environmental enrichment provision in Australian intensive livestock industries and industry-specific quotations

Practical consideration	Quotations from industry survey	Industry
Waste disposal method	*“Foreign objects entering the litter stream at clean out time”*	Chicken meat
	*“Sand couldn’t be composted”*	Chicken meat
	*“Won’t work with effluent system”*	Pork
Biosecurity standards	*“Difficult to clean between batches [of birds]”*	Chicken meat
	*“Not practical as mites penetrate wood”*	Egg
Obligatory management practices	*“.…. impractical with very large broiler sheds – distributing and re-distributing for pickup”*	Chicken meat
	*“risk for feed in crop at processing”*	Chicken meat
	*“may get floor eggs”*	Egg
	*“risk of unbalancing diet and risk of under-consuming anti-coccoidal medications”*	Chicken meat
	*“Block slats….”*	Pork
Accessibility	*“Access to this substrate is a problem”*	Chicken meat
	*“Rice hulls are increasingly difficult to source”*	Pork

### Case studies from the scientific literature

We apply the results from common environmental enrichment items reported in the scientific literature for meat chickens, gestating sows, feedlot beef cattle and laying hens, (sand, straw, brushes and perches, respectively) as an example of how our proposed framework may be utilised to communicate the benefits of each environmental enrichment to livestock welfare (see Table S4).

The scientific literature suggests, based on the available evidence, that sand is a form of ‘pseudo-enrichment’ for meat chickens. Meat chickens prefer sand over other substrates, but the use of sand is not sustained nor is there any evidence of improved biological function or positive welfare balance, unless it is paired with a suite of other enrichment items. When sand is provided with PECKStone^TM^ (Vilofloss, Federicia, Denmark), perches and novel objects there is evidence that meat chickens’ mood (and therefore positive welfare balance) is improved (Table S4). This enrichment programme would therefore be considered ‘environmental enrichment for a positive welfare balance.’

Literature reporting the effects of nesting materials provided to pregnant sows around the time of parturition show that hessian sacks (although used short-term) are a form of ‘pseudo-enrichment’ with no improvements to biological functioning. Conversely, straw (depending on how and when it is offered) generally improves biological functioning, evidenced by a reduction in glucocorticoid concentrations and improved reproduction (Table S4). With nesting enrichments only provided around parturition, it is unlikely that these effects will result in long-term positive welfare balance of the sow’s life, however this remains unknown. Based on the current available literature, the provision of straw to sows close to partition would be classified as an ‘enrichment for meeting basic needs.’

The scientific literature suggests that perches (in isolation) enrich laying hen environments for *pleasure.* Hens are motivated to access perches and their use is sustained until the end of the production cycle, but there is no evidence that they improve biological functioning or long-term positive welfare balance (Table S4).

There is a lack of investigations that utilise both short- and long-term assessments of suffering, pleasure and welfare balance after the provision of environmental enrichment (Table S4). We propose that future investigations of environmental enrichment study suffering, pleasure and welfare balance throughout the animal’s lifecycle, as outlined in Table 2. Applying the framework to the current literature, the true impact of environmental enrichments on animal welfare may be underestimated, as it is clear from Table S4, that long-term assessments of positive welfare balance (i.e. stress resilience and positive mood) are under-researched and under-reported.

## Discussion

Baseline environments are likely to affect the impact of enrichment provision on animal welfare. Animals housed in barren environments are likely to show disrupted biological functioning (Beattie *et al.* 2000) and a pessimistic mood (Douglas *et al.* 2012) and therefore some items may evoke greater improvements to welfare compared to the same enrichment item in a more complex environment. For example, deprivation of essential stimuli leads to sensitivity to rewards (Van der Harst *et al.* 2003), as such the experience and value of an environmental enrichment will change as standard/baseline environments do. This is a critical component that our framework has considered, such that we focus on the magnitude of the improvements to animal welfare after the provision of environmental enrichment item or programme, that can describe the improvements even as minimum housing of production animals change. As such, this framework focuses on continual *improvements* to animal welfare after the provision of enrichment which negates a ‘one enrichment fits all’ solution (e.g. ‘chains’ are enriching to pigs). As continual improvements are made to the standard environments of intensively housed animals, this framework offers flexibility to assess the effectiveness of enrichments when they are added to the ‘standard’ or ‘typical’ housing environment. This framework does not dictate the standard intensive environment animals *ought* to be housed in, this has (and will likely continue) to change as social norms evolve. Rather, we provide a framework to indicate the relative animal welfare *improvements* after the provision of enrichment that can be used as evidence provided to consumers, regulatory bodies and producers. Therefore, this framework should be used to indicate the relative improvement from the ‘industry standard’ relative to time, place and market.

The framework presented in this study provides a guide for considering the effectiveness of environmental enrichment to improve animal welfare at the group level and does not account for individual differences between animals. Use (and therefore effectiveness) of enrichments may be related to individual differences caused by variation in previous experience, temperament, or genetics (Widowski & Duncan 2000) which will result in heterogenous improvements to animal welfare within the group, but an overall improvement for the flock, herd or drove.

Our survey data indicated that of the enrichments identified in the literature search to improve animal welfare (n = 67 enrichment items/programmes, supplementary material) only 33% on average were currently utilised by industry (6% pork industry; 34% egg industry; 56% chicken meat industry). These data may suggest a disconnect between research and industry; a poor response rate makes our results inconclusive but similar findings have been reported elsewhere (Peden *et al.* 2018). This may be related to concerns with practicality (actual or perceived) or economics. Indeed, the survey showed that costs were industry’s greatest perceived barrier to implementing enrichments, which has also been recently reported (Peden *et al.* 2021). As highlighted by our survey, the assessment of economic benefits and costs for effective and practical enrichments is affected by familiarity with the enrichments as well as their implementation and maintenance costs (e.g. material). Broader benefits from providing animal enrichments (e.g. access to retailers, gaining/maintaining social licence) may not generate an immediate economic return but may contribute to a competitive advantage of a livestock production business which can be difficult to value in an economic benefits and costs assessment. Further, the time lag between input and benefit may make it difficult for producers to recognise the positive impact of enrichment provision on the productivity and sustainability of their enterprise. This framework provides a structure to discuss the links between animal welfare outcomes and productivity improvements and premium price returns.

### Animal welfare implications

This body of work provides a framework for livestock producers to ensure that the provision of enrichments is effective, in the sense that they are feasible and economical to implement and lead to actual improvements to animal welfare. As such, this framework focuses on the *outcome* of enrichment provision rather than the *intent.* Changing the narrative from simply ‘ticking a box’ when providing enrichments also provides the flexibility required for continual improvements to animal welfare, even when baseline (industry standard) environments evolve.

## Conclusion

This paper proposes a framework to re-define and classify effective environmental enrichments. It incorporates four welfare outcome categories which are science-based and may be utilised in a variety of contexts including, to inform on the development of animal welfare legislation, assurance programmes, product differentiation and labelling. The impact of the enrichment is the focus rather than the intent and our approach aims to ensure a continued improvement to production animal welfare. We highlight knowledge gaps in the scientific literature regarding livestock enrichment programmes, that would benefit from both short- and long-term assessments to measure suffering, pleasure and welfare balance. The inclusion of practicality and economics promotes interactions between researchers, regulatory bodies and industry to develop proposed enrichments which are industry-relevant and feasible to implement. This framework can help stakeholders communicate the effects of their enrichment programmes on animal welfare to the public and consumers and create genuine improvements to animal welfare.

## Supplementary material

For supplementary material accompanying this paper visit https://doi.org/10.1017/awf.2023.5. click here to view supplementary material
