# An effective environmental enrichment framework for the continual improvement of production animal welfare – ERRATUM

**DOI:** 10.1017/awf.2023.23

**Published:** 2023-03-01

**Authors:** Peta S Taylor, Peggy Schrobback, Megan Verdon, Caroline Lee

The publisher apologises that within the author contributions section the following text incorrectly included in the final paper:

Please confirm whether the Author Contributions section is correct.

This line has now been removed from the article.

## References

[r1] Taylor P, Schrobback P, Verdon M and Lee C 2023 An effective environmental enrichment framework for the continual improvement of production animal welfare. Animal Welfare, 32, E14. doi:10.1017/awf.2023.5 38487434 PMC10936304

