# Supplementary material for: An effective environmental enrichment framework for the continual improvement of production animal welfare
Source: Anim Welf. 2023 Feb 14;32:e14. doi: 10.1017/awf.2023.5 (PMC10936304; doi:10.1017/awf.2023.5)
Supplement: Supplementary file 1 [file awfsup.zip › S0962728623000052sup001.pdf]

Survey: Assessing the practicality, cost and benefits of effective environmental enrichment programs

1. Which industry do you represent? Please choose only one - you may complete the survey a second time if you wish to provide feedback on another industry
   - Beef
   - Dairy
   - Meat chicken
   - Laying hen
   - Pork
   - Sheep
2. What is your role?
   - Producer
   - Peak body representative
   - Welfare officer
   - Veterinarian
   - Other
3. How many years have you been working in the industry?
4. In which state is your business located?
5. Please state the relative size of your enterprise

Pork industry
- Pig keeper (< 8 sows)
- Small holder (8-50 sows)
- Small commercial (51-150 sows)
- Medium commercial (151-500 sows)
- Large commercial (500-1000 sows)
- Very large commercial (1000+ sows)

Beef cattle
- Small (< 5,000 head)
- Medium (5,000 - 10,000 head)
- Large (10,000 - 20,000 head)
- Very large (20,000 + head)

Sheep
- Small (< 5,000 head)
- Medium (5,000 - 10,000 head)
- Large (10,000 - 20,000 head)
- Very Large (20,000 + head)

Meat chickens
- Very small (< 30,000 chickens)
- Small (< 6 sheds: 30,000 - 50,000 chickens/shed)
- Medium (7-12 sheds: 30,000 - 50,000 chickens/shed)
- Large (12 + sheds: 30,000 - 50,000 chickens/shed)

Laying hens
- Hobby (< 1000 birds)
- Medium (1,000 - 24,900 birds)
- Large (25,000 + birds)

What time of housing systems do you utilise
- Aviary
- Free-range Aviary
- Conventional cage
- Flat deck (barn)
- Free-range flat deck
- Other ___________

6. Please indicate if you currently provide any of the following enrichments

Pork industry	Beef cattle	Sheep	Laying hens	Meat chickens
Peat moss	Shade - Steel ion/sheets	Shade - corrugated iron	UV-A light	Bedding - rice hulls
Hay straw (long)	Shade - polyethylene fabric	Phalaris	Wooden perch	Bedding - wood shavings
Hay straw (chopped)	Shade - Corrugated iron sheets	Straw hay bales	Perching structure with solid vertical panels	Bedding - recycled paper roll
Barley straw (long)	Shade - 80% UV block shade cloth	Shrubs	Artificial turf	Bedding - hemp waste
Barley straw (chopped)	Shade - 99% UV block shade cloth	Trees		Substrate - peat moss
Wheat straw (long)	Brushes fixed horizontal	Barley straw	Chopped straw	Substrate - sand
What straw (chopped)	Brushes fixed L shaped	Cereal straw	Whole straw bedding	Foraging materials - whole wheat
Lucerne Hay	Brushes automated (i.e. motion activated)	Wheat straw	Wood shavings	Foraging materials - mealworms
Shredded newspaper	Drum covered in turf	Sand bedding	Pecking stones	Foraging materials - oat hulls
Sawdust	Drums that contain hay	Straw bedding	Dust bath with peat moss	Foraging materials - feed pellets
Straw dispenser		Platforms	Dust bath with sand	Haybales
Wood shavings		Ramps	Grain	String
Rubber mats			Propylene ropes	Natural light
Rope			Maize silage	Moving coloured lights
Sand flooring			Barley-pea silage	Perch
Jute sacks			Carrots	Platform
Tree branches			Pellet feed	Short dark and light intervals to mimic maternal environment
Sisal ropes			Mash feed	
			Dark brooders	Lazer lights
			Tennis balls in feeders	Toys that are replaced frequently (e.g. plastic balls, plastic bottles, mirrors) changed every three days
			String	
			Visual contact with humans	
			Haybales	
			Plastic clear box	
			Wooden board with stones	
			Porous concrete block	
			Abrasive strip	
			Metal plate with holes	
			Rubber mats	
			Video images	
			Music	
			Wooden blocks covered in tin foil, tissue paper and felt paper	
			Novel objects (balls, coloured drawings) changed every 3 days	

7. Please note any other enrichments you currently provide that is not listed above and why you provide it. Alternatively, if you do not provide any enrichments please tell us why you don't.
8. Please provide an evaluation of the practicality of specific enrichments (e.g., their feasibility to be added to farming systems considering aspects such as infrastructure need, time, effort, and other implication for the system) by ranking each enrichment on the sliding scale from not at all practical (0) to very practical (100)
9. From your perspective, please rank the following potential motives for farmers to implement environmental enrichments that aim to improve animal welfare. Hover the mouse over the options and drag each option into your rank from (1) = most important to (8) = least important

    To improve animal health and farm profitability
    Increase the farm and/or industry's social license to operate
    To meet the standards of accreditation agencies
    Peer pressure
    Pressure from consumers
    Pressure from retail sectors
    Pressure from lobby groups
    Other, please specify

10. From your perspective, please rank the following potential barriers for farmers not to implement enrichments? Hover the mouse over the options and drag each option into your rank from (1) = most important to (6) = least important

    Costs of implementation, including time and communication
    Pressure from stakeholders (e.g. consumers, retailers, lobby groups) is not high enough
    Long term feasibility of enrichments is uncertain
    No regulation that enforces adoption of enrichments
    Lack of knowledge about the costs and benefits about enrichments that improve animal welfare
    Other, please specify

Survey responses asking whether specific enrichments were currently being, or had previously, been utilised from representatives from the Australian meat chicken, egg and pork industries.

**Industry**	**Enrichment**	**Utilised**
Meat chicken industry	Bedding - rice hulls	Y
	Bedding - wood shavings	Y
	Bedding - recycled paper roll	Y
	Bedding - hemp waste	N
	Substrate - peat moss	N
	Substrate - sand	N
	Foraging materials - whole wheat	Y
	Foraging materials - mealworms	N
	Foraging materials - boat hulls	N
	Haybales	Y
	String	Y
	Natural light	Y
	Moving coloured lights	N
	Short dark and light intervals	Y
	Laser lights	N
	Toys (e.g. plastic balls, plastic bottles, mirrors)	N
	Perch	Y
	Platform	Y
Egg industry	UV-A light	N
	Wooden perch	N
	Perching structure with solid vertical panels	Y
	Artificial turf	Y
	Ramps	Y
	Chopped straw	Y
	Whole straw bedding	Y
	Wood shavings	Y
	Pecking stones	Y
	Dust bath with peat moss	N
	Dust bath with sand	N
	Grain	N
	Propylene ropes	N
	Maize silage	N
	Barley-pea silage	N
	Carrots	N
	Dark brooders	N
	Tennis balls in feeders	N
	String	N
	Visual contact with humans	Y
	Haybales	Y
	Plastic clear box	N
	Plastic clear box	N

Industry	Enrichment	Utilised
Egg industry Cont.	Wooden blocks covered in tin foil, tissue paper and felt paper	N
	Wooden board with stones	N
	Porous concrete block	N
	Abrasive strip	N
	Metal plate with holes	Y
	Rubber mats	Y
	Novel objects (balls, coloured drawings) changed every 3 days	N
	Video images	N
	Music	N
Pork industry	Peat moss	N
	Hay straw (long)	N
	Hay straw (chopped)	N
	Barley straw (long)	N
	Barley straw (chopped)	N
	Wheat straw (long)	N
	What straw (chopped)	N
	Lucerne Hay	N
	Shredded newspaper	N
	Sawdust/rice hulls	N
	Straw dispenser	N
	Wood shavings	N
	Rubber mats	N
	Sisal Rope	Y
	Sand flooring	N
	Straw bakes/jute sacks	N
	Tree branches	N

Motives and barriers for implementation of animal enrichments (average rating). Rating scale from 1 for most important to 6 or 8 for indication least important motive or barrier, respectively. Results are reported as mean rank and standard deviation. A standard variation of zero and (*) indicates that only one response was recorded.

**Motives**	**Chicken meat**	**Pork**	**Egg**	**Feedlot cattle/dairy**	**Feedlot sheep**
To improve animal health and farm profitability	1.00 ± 0.0	3.25 ± 2.2	1.00 ± 0.0*	2.00 ± 0.0*	2.00 ± 0.0*
Increase the farm and/or industry's social license to operate	3.25 ± 1.3	1.50 ± 1.0	2.00 ± 0.0*	3.00 ± 0.0*	1.00 ± 0.0*
To meet the standards of accreditation agencies	3.00 ± 1.4	4.75 ± 1.7	3.00 ± 0.0*	8.00 ± 0.0*	6.00 ± 0.0*
Peer pressure	5.75 ± 1.3	5.50 ± 1.3	5.00 ± 0.0*	4.00 ± 0.0*	7.00 ± 0.0*
Pressure from consumers	5.00 ± 1.4	4.00 ± 1.2	6.00 ± 0.0*	5.00 ± 0.0*	4.00 ± 0.0*
Pressure from retail sectors	3.50 ± 1.3	3.00 ± 2.0	4.00 ± 0.0*	6.00 ± 0.0*	5.00 ± 0.0*
Pressure from lobby groups	6.50 ± 0.6	6.00 ± 1.4	7.00 ± 0.0*	7.00 ± 0.0*	3.00 ± 0.0*
Other	8.00 ± 0.0	8.00 ± 0.0*	8.00 ± 0.0*	1.00 ± 0.0*	8.00 ± 0.0*
**Barriers**	**Chicken meat**	**Pork**	**Egg**	**Feedlot cattle/dairy**	**Feedlot Sheep**
Costs of implementation, including time and communication	1.2 ± 0.5	1.25 ± 0.5	1.00 ± 0.0*	2.00 ± 0.0*	1.00 ± 0.0*
Pressure from stakeholders (e.g. consumers, retailers, lobby groups) is not high enough	3.8 ± 1.3	2.75 ± 1.5	5.00 ± 0.0*	5.00 ± 0.0*	4.00 ± 0.0*
Long term feasibility of enrichments is uncertain	3.4 ± 1.1	3.00 ± 0.0	4.00 ± 0.0*	6.00 ± 0.0*	2.00 ± 0.0*
No regulation that enforces adoption of enrichments	4.0 ± 1.2	4.75 ± 0.5	3.00 ± 0.0*	4.00 ± 0.0*	5.00 ± 0.0*
Lack of knowledge about the costs and benefits about enrichments that improve animal welfare	2.6 ± 1.1	3.25 ± 1.5	2.00 ± 0.0*	1.00 ± 0.0*	3.00 ± 0.0*
Other	6.0 ± 0.0*	6.00 ± 0.0*	6.00 ± 0.0*	3.00 ± 0.0*	6.00 ± 0.0*
