# Supplementary material for: An effective environmental enrichment framework for the continual improvement of production animal welfare
Source: Anim Welf. 2023 Feb 14;32:e14. doi: 10.1017/awf.2023.5 (PMC10936304; doi:10.1017/awf.2023.5)
Supplement: Supplementary file 1 [file awfsup.zip › S0962728623000052sup002.pdf]

Table S4. Case studies for proposed environmental enrichment (EE) items identified in the scientific literature with reported outcomes for biological functioning, short-term hedonic positive states, sustained use and indicators of overall positive welfare balance. WS indicates Wood shaving litter; RPR indicates Recycled Paper Roll litter; RH indicates Rice hull litter; FC indicates Farrowing crate; LWL indicates Large White × Landrace cross; LMP indicates Landrace x mini pigs; DMP indicates Duroc x mini pigs; Landrace x large whiteBC indicates British continental crossbreed; B indicates British breed; JB indicates Japanese Black; C indicates Charolais cross. – indicates that the factor was no reported or unclear.

Species	Proposed EE	Details of EE	Age/ Duration	Breed	EE density	Baseline housing	Short term assessment		Long-term assessment		Citation	Framework classification *based on available information*
							**Biological functioning**	**Hedonic positive affect**	**Sustained use**	**Positive mood/stress resilience**		
Meat chicken	Sand	17.8cm sand bedding	0–50d	Ross308	2cm deep bedding	Pen; WS; RPR; RH	↔[1]	↑[2]	↓[†]		Toghyani et al. (2010)	
		2 x sand trays	0–37d	Ross308	1×(67×46×4cm)/40 head 1×(37×36×4cm)/40 head	Pen; WS		↑[2]	↓[†]		Arnould et al. (2004)	***Psuedoenrichment****
		Sand pit	0-42d	Cobb500	3.8 × 8.9 cm pit with sand 2.5cm deep	Pen; WS		↑[2]	↓[†]		Shields et al. (2005)	
Sows prior to partirition	Hessian sack	Hessian sacks	Multiparous	LWL	2 × hessian sacks fixed to bars	FC	↔[3, 4, 5]				(Plush et al., 2011)	***Psuedoenrichment****
Sows prior to partirition	Straw	Chopped straw	Multiparous	LWL	2 kg straw refreshed daily until farrowing	FC	↑[3, 4, 5]				(Plush et al., 2011)	
		Deep straw and additional space	Multiparous	LWL	3.4 m^2/sow	Pen	↑[3, 1]				(Merlot et al., 2017)	
		Deep straw	Primiparous/ Multiparous	Landrace x Large white	3.4 m^2/sow	Pen	↑[3 ,1]				(Merlot et al., 2019)	
		Straw in box	Multiparous	DanBred	Replenished as needed	FC	↑[4]				(Martins et al., 2021)	
		Long barely straw and chain	Primiparous/ Multiparous	PIC Camborough	1.5 kg straw each morning	Pen & feeding stall	↔[3]				(Spoolder et al., 1996)	***Enrichment for meeting basic needs***
		Hay straw	-	TopGen Afrodite	Replaced daily	Pen	↔[3]				(Tatemoto et al., 2019)	
		Straw	Multiparous	LMP; DMP; LWL	3.5 kg daily	FC	↑[3, 6]	↑[7]			(Wang et al., 2020)	
		Loose straw	Multiparous	Swedish Landrace x Yorkshire	15 – 20 kg 2 days before farrowing & 2 kg daily 5 d post farrowing then 0.5-1 kg daily	Pen	↑[4]				(Westin et al., 2015)	
Beef cattle	Brush	L shaped brush	64d	B, BC	1/27 head	Feedlot			↑		Meneses et al. (2019)	
		A horizontal and vertical attached brush	178d	JB	2 (50×10×110cm) /4 head	Feedlot	↑[8]		↑		Ninomiya (2019)	***Enrichment for meeting basic needs***
		L shaped brush	64d	B	5m^2/head	Feedlot	↔[2, 9]		↑		(Park et al., 2020)	
		Brush	256d	B	1/18head	Feedlot	↔[9]		↑		(Park et al., 2019)	

		Arched moveable brush	22d	C	2×(1.5m H)/10 head	Feedlot & scent devices		↑[2]			(Wilson et al., 2002)	
Layer hen	Perch	Rounded metal perches 32mm diameter	Pullet	White Leghorn	3.2 – 10.2 cm/hen	Conventional cage	↔[10, 9]				Enneking et al. (2012)	
		Rounded wood 30mm diameter	Adult	Hyline	-	Conventional cage	↔[3]				Barnett et al. (2009)	
		Rounded hardwood 40 mm diameter	Adult	White Lomann	22cm/hen	Conventional cage	↔[4]				Frediani et al. (2019)	***Enrichment for pleasure***
		Round metal 32 mm diameter	Adult	Hy-Line	16.9cm/hen	Conventional cage	↔[4]				Hester et al. (2013)	
		Round metal perch (3.18 cm diameter)	Adult	White Lomann	15 cm/hen	Commerical aviary		↑[11]			Campbell et al. (2016)	
		1 m wide, 23 and 43cm high	Adult	Lohmann leghorn	-	Pen, tested in cages			↑[†]		Olsson and Keeling (2002)	
Meat chicken	Sand & complexity	Sand filled dust bath, PECK stone, 3 × PVC pipe perches and novel objects	0–50d	Ross708	Dust bath: 180×91×10cm Perches: 183×31×9cm; 1.9cm diameter	Pen & WS				↑[12†] ↑[13†]	Anderson et al. (2021a) Anderson et al. (2021b)	***Enrichment for positive welfare balance***

[1] Immunefunction, Antibody titre response, spleen and bursa of fabricius weight
[2] Preference
[3] Glucocorticoid concentrations
[4] Reproduction, litter size, stillborn, weaned
[5] Immunefunction colustrum IgG
[6] Prolactin
[7] Oxytocin
[8] Prevalence of visceral disease
[9] Body weight
[10] Adrenal gland weight
[11] Behavioural demand
[12] Attention bias test
[13] Stress resilience; startle refex & autonomic response to aversive stimli
[†] End of production cycle/life assessment
